# Why do they die? Comparison of selected aspects of organ injury and dysfunction in mice surviving and dying in acute abdominal sepsis

**DOI:** 10.1186/s40635-015-0048-z

**Published:** 2015-04-07

**Authors:** Susanne Drechsler, Katrin M Weixelbaumer, Adelheid Weidinger, Pierre Raeven, Anna Khadem, Heinz Redl, Martijn van Griensven, Soheyl Bahrami, Daniel Remick, Andrey Kozlov, Marcin F Osuchowski

**Affiliations:** Ludwig Boltzmann Institute for Experimental and Clinical Traumatology, Trauma Research Center of AUVA, Donaueschingenstrasse 13, Vienna, 1200 Austria; Department of Anaesthesia, University Hospital Regensburg, Franz-Josef-Strauß-Allee 11, Regensburg, 93053 Germany; Department of Pathology and Laboratory Medicine, Boston University School of Medicine, Boston, MA 02118 USA; Current address: ViruSure GmbH, University of Veterinary Medicine Vienna, Veterinärplatz 1, Vienna, 1210 Austria; Current address: Experimental Trauma Surgery, Clinic for Trauma Surgery, Klinikum Rechts der Isar, Technical University Munich, Ismaninger Straße 22, Munich, 81675 Germany

**Keywords:** Inflammation, Survival, Outcome prediction, Body temperature, Organ failure

## Abstract

**Background:**

The mechanisms of sepsis mortality remain undefined. While there is some evidence of organ damage, it is not clear whether this damage alone is sufficient to cause death. Therefore, we aimed to examine contribution of organ injury/dysfunction to early deaths in the mouse abdominal sepsis.

**Methods:**

Female OF-1 mice underwent either medium-severity cecal ligation and puncture (CLP-Only) or non-lethal CLP-ODam (CLP with cisplatin/carbontetrachloride to induce survivable hepatotoxicity and nephrotoxicity). In the first experiment, blood was collected daily from survivors (SUR; CLP-Only and CLP-ODam groups) or until early death (DIED; CLP-Only). In the second experiment (CLP-Only), early outcome was prospectively predicted based on body temperature (BT) and pairs of mice predicted to survive (P-SUR) and die (P-DIE) were sacrificed post-CLP. The overall magnitude of organ injury/dysfunction was compared in retrospectively and prospectively stratified mice.

**Results:**

At day 7 post-CLP, survival in CLP-Only was 48%, while CLP-ODam was non-lethal. In CLP-Only mice within 24 h of death, urea increased to 78 (versus 40 mg/dl in SUR), ALT to 166 (vs. 108 U/l), LDH to 739 (vs. 438 U/l) and glucose declined to 43 (vs. 62 mg/dl). In CLP-ODam, hypoglycemia was exacerbated (by 1.5-fold) and ALT and LDH were 20- and 8-fold higher versus DIED (CLP-Only) mice. In CLP-Only, predicted deaths (P-DIE) were preceded by a significant rise only in cystatin C (268 vs. 170 ng/ml in P-SUR) but not in creatinine and troponin I. Respiratory function of mitochondria in the liver and kidney of P-SUR and P-DIE CLP-Only mice was not impaired (vs. controls) and ATP level in organs remained similar among all groups. Histologic injury scores in the liver, kidney, heart and lung showed no major disparities among dying, surviving and control mice.

**Conclusions:**

In CLP-Only mice, although the deregulation of parameters indicative of organ injury/dysfunction was greater in dying versus surviving mice, it never exceeded the changes in surviving CLP-ODam animals, and it was not followed by histopathological damage and/or mitochondrial dysfunction. This shows that interpretation of the contribution of the organ injury/dysfunction to early deaths in the CLP model is not straightforward and depends on the pathophysiological origin of the profiled disturbances.

**Electronic supplementary material:**

The online version of this article (doi:10.1186/s40635-015-0048-z) contains supplementary material, which is available to authorized users.

## Background

Sepsis syndromes belong to the most challenging conditions in contemporary critical care. In young immuno-competent subjects, sepsis onset is typically associated with hyperinflammation reflected by a strong and simultaneous release of pro- and anti-inflammatory mediators in the blood [1]. In contrast, in immune-compromised patients, e.g., older, with comorbidities and/or after major trauma, the ‘cytokine storm’ is frequently replaced by a severe immunosuppression from the very onset of the disease [1]. Each of the above reactions, however, can disrupt the functional homeostasis of vital organs including the lungs, heart, liver and kidney [2]. As sepsis evolves, these initial perturbations may, especially in the case of an over-excessive cytokine burst, quickly evolve into the multiple organ dysfunction syndrome (MODS, also referred to as multiple organ failure or MOF). In the ICU patients with sepsis, MODS frequently develops as a secondary complication with fatal outcome [3]: e.g., in the PROWESS trial, MODS was one of the three most common causes for sepsis-related death [4,5].

However, despite its lethality, the exact pathogenesis of MODS remains unclear [6]. While an early-onset (days 0 to 3) MODS secondary to acute insults such as sepsis and trauma has been observed in patients, MODS typically develops in a more protracted fashion and over the course of several days [7-11]. The exact sequence of the early events that lead to organ failure and its contribution to acute deaths await in-depth characterization [2]. For example, it was demonstrated that non-infected traumatized patients without signs of MODS and patients with early-onset MODS demonstrated the same risk of lethal outcome [2,9]. In septic patients, the most recent in-depth analysis demonstrated that MODS is often a key contributor to mortality but not through a rapid and overt failure but rather through a protracted process of the gradual decline in the organ function [5]. Similarly, in septic children, early-onset MODS is associated with much milder morbidity and mortality compared to secondary MODS [12]. This evidence invites a speculation that in young and uncomplicated septic subjects, the life-threatening impairment of multiple organs (i.e. an overt MOF stage) will not be reached immediately but at later time points. Relatively minimal histopathological changes (and/or lack thereof) in organs of acutely dying septic patients indirectly support such a notion: in the recent studies by the Hotchkiss group, autopsies performed on patients who died in the intensive care unit (ICU) detected only trivial signs of apoptosis/necrosis in the heart, kidney, liver and lung [13,14]. Notably, findings from few existing (clinically relevant) mouse models of trauma and sepsis parallel, at least partly, the aforementioned clinical findings. For example, trauma/hemorrhage followed by cecal ligation and puncture (CLP)-sepsis in the mouse did not induce marked changes in the lung and kidney morphology for up to 96 h post-CLP [15] and pulmonary injury did not contribute to death in another acute CLP study [16].

Given that the role of MODS in acute septic deaths is not fully understood, this study aimed to investigate how the magnitude and dynamics of deregulated homeostasis in major organs is related to early outcome in young outbred mice suffering from polymicrobial abdominal sepsis. Specifically, we chose several most commonly used parameters characterizing selected aspects of cellular injury and/or function of vital organs and compared them among CLP mice either dying or surviving the acute phase of sepsis and surviving septic animals with added organ damage.

## Methods

### Animals

Female, 3- to 4-week-old, outbred OF-1 mice (Himberg, Austria) were used for all experiments (the exact *n*/group distributions are specified in legends to the figures). After arrival, all animals were allowed to acclimatize to their new environment for at least 1 week. Mice were kept together in groups of five per type III cage, housed on a 12 h light-dark diurnal cycle with controlled temperature (21°C to 23°C) and provided with standard rodent diet and water *ad libitum* throughout all experiments. Cages were enriched with houses, wood wool for nesting as well as wooden boards, tunnels and small blocks for gnawing (Abbedd Lab & Vet Service, Vienna, Austria) to facilitate natural behavior prior to and throughout the experimentation.

### Ethics statement

All animal procedures were approved by the Viennese (Austria) legislative committee (Animal Use Proposal Permission no 007602/2007/10) and conducted according to the National Institutes of Health guidelines.

Given the severity of our study, we employed a maximally diligent observation of all mice to minimize suffering within the frames of the experimental design. All mice enrolled in the study were kept in the institute's small in-house animal facility to enable optimal and frequent monitoring: the overall health status was checked by trained professionals (i.e. DVMs and/or MDs) at least three times per day and more (typically every 2 to 3 h) whenever an animal's condition deteriorated (defined by, among other parameters, decreased activity, progressing hypothermia, rapid weight gain) [17,18].

Given that the objective of our study was to compare the magnitude and dynamics of organ injury/dysfunction in early murine sepsis between acutely dying and long-term surviving mice, the time of septic death was a critical endpoint. In survival studies, as recommended by Nemzek et al*.* [18], we followed a precise, empirically established set of guidelines that offers a feasible window of opportunity (due to the frequent monitoring) for euthanasia in moribund mice at the earliest possible time simultaneously retaining the 100% certainty of their impending demise (to eliminate the type I error). Specifically, mice were killed only upon signs of imminent death (i.e. inability to maintain upright position/ataxia/tremor and prolonged/deep hypothermia and/or agonal breathing) by using deep inhalation anesthesia (isoflurane) followed by an overdose of barbiturate (thiopental, Thiopental Sandoz®; Sandoz International GmbH, Holzkirchen, Germany).

### Sepsis model

All mice were subjected to CLP surgery according to the original protocol by Wichterman et al*.* [19] with specifications made elsewhere [20]. We relied on the CLP model given that it closely mimics many aspects of the inflammatory, hemodynamic and metabolic responses observed in human sepsis originating from the abdominal compartment [1,21]. Furthermore, the presence of infectious focus (i.e. necrotic cecum) in CLP mice is in line with the clinical scenario given that a continuous septic focus is frequently present in septic patients despite attempts at appropriate source control [22]. We purposely used healthy, young (4 weeks old) outbred mice: this allowed us (1) to directly examine the link between the development of organ injury/dysfunction in the consistently robust hyperinflammatory environment [23] and (2) to avoid strong confounding factors such as age [24] and comorbidities [25].

Two CLP protocols were used in the study: (1) medium-severity (18G needle; henceforth referred to as CLP-Only mice, *n* = 83) to reach an approximate 30% to 50% mortality and (2) low-severity (23G needle; used only in the CLP-ODam subgroup of mice; the ODam component explained below) protocol with mortality typically not exceeding 10% by day 5 post-CLP. In brief, after opening the abdominal cavity via midline laparotomy, the cecum was exposed, ligated and punctured twice. After repositioning of the cecum, the abdomen was then closed with two single button sutures and Histoacryl® skin adhesive (B. Braun, Aesculap, Tuttlingen, Germany). Starting 2 h post-CLP, all mice received subcutaneous wide-range antibiotic therapy (25 mg/kg imipenem, Zienam®; MSD, Lucerne, Switzerland) and fluid resuscitation (1 ml Ringer's solution) with analgesia (0.05 mg/kg buprenorphine, Buprenovet®; Bayer, Maharashtra, India) twice daily (in approximately 12 h intervals) for five consecutive days post-CLP.

### Non-lethal model of CLP and CCl_4_/cisplatin

To induce an acute but survivable hepatocyte injury and kidney dysfunction in CLP mice (with low mortality; see the next section), a single dose of carbon tetrachloride (CCl_4_; 0.3 μl/g of body weight) and cisplatin (1 μg/g of body weight) was administered into the open abdominal cavity at the time of CLP surgery; we will henceforth designate this group as CLP-ODam. The subjective ODam acronym signifies the presence of tissue injury caused to the liver and kidney by exposure to CCl_4_/cisplatin, although this is simultaneously accompanied by functional deterioration of those organs [26-28]. Of note, the injury and dysfunction recorded in the CLP-ODam mice (although exacerbated compared to CLP-Only) are not equivalent to an overt organ failure post-CLP.

Three CLP-ODam mice were sampled at 24, 48 and 72 h post-CLP and were followed for 7 days after CLP (and euthanized on day 8 post-CLP) to ensure the CLP-ODam setup was not lethal in the acute phase of sepsis. CCl_4_ is a potent hepatotoxin commonly used to induce cirrhosis in mouse models of liver disease [29,30], while cisplatin is a chemotherapeutic agent with strong nephrotoxic side effects [31] utilized in mouse models of acute kidney injury (AKI) [26,32]. Furthermore, both agents trigger a strong release of inflammatory cytokines (e.g. TNFα, IL-1β, IL-6) [27,28]. We chose the liver and kidney as the target organs given that their injury/dysfunction is commonly investigated in septic patients and CLP mice [6,33,34]. Of note, after selection of the non-lethal CCl_4_/cisplatin dose, the number of mice enrolled to the surviving CLP-ODam group was kept to minimum (i.e. three, but sampled sequentially over 3 days) given the severe burden of the combined CLP/CCl_4_/cisplatin challenge.

A complete illustration of severe CCl_4_/cisplatin-induced damage to the liver and kidney is provided by previous studies [6,26-28,30,32]; our objective was not an in-depth characterization of organ damage in the CLP-ODam mice but to use the obtained measurements (of the selected circulating parameters in the first experiment) for comparison purposes. Therefore, in agreement with the ARRIVE guidelines [35] and 3R (Reduce, Replace, Refine) principle, we elected not to include a separate CLP-ODam group in the second experiment, in which additional circulating markers of organ dysfunction were analyzed (i.e. cystatin C, troponin I) and histopathological comparison of organ injury was performed (based on the prospective outcome stratification; see below).

### Experimental setup and outcome stratification protocols

**Figure 1:**
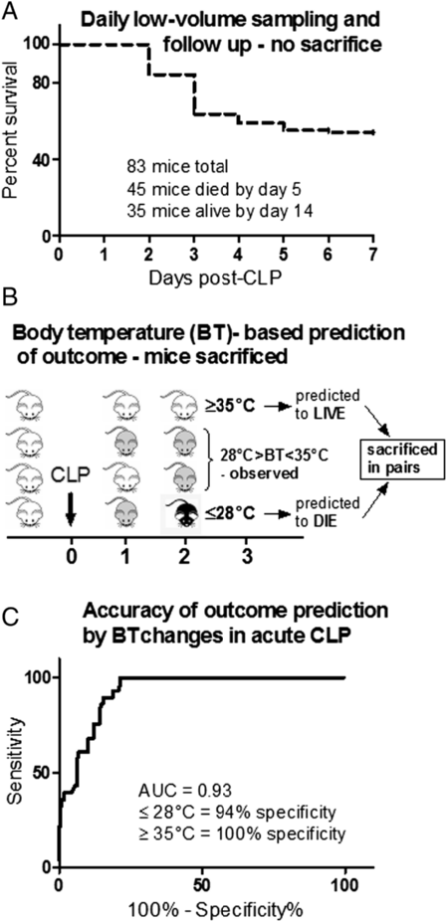
**Survival curve and outcome prediction based on body temperature. (A)** Seven-day mortality in 4-week-old OF-1 mice subjected to CLP-Only and daily low-volume blood sampling for days 1 to 5 post-CLP. **(B)** Experimental setup for prospective BT-based outcome prediction. A septic mouse was predicted to die (P-DIE) whenever its BT dropped below 28°C within days 1 to 5 post-CLP. Upon detection, each P-DIE mouse was always sacrificed with a matching predicted to survive (P-SUR) mouse (i.e. with BT ≥35°C). **(C)** ROC curve and corresponding area under the curve (AUC) for the prediction of outcome based on BT in acute CLP sepsis (days 1 to 5 post-CLP). BT ≤28°C identified dying mice with 94% specificity, while a BT ≥35°C identified surviving mice with 100% specificity. The BT cutoffs for outcome prediction utilized in this study were predetermined based on the internal BT measurement database compiled in previous studies.

Depending on the needle gauge, each CLP mouse was assigned to either (1) CLP-Only or (2) CLP-ODam. In the CLP-Only group, two outcome stratification approaches were applied. In the first experiment, mice were monitored for 14 days for outcome and retrospectively stratified into those that died (henceforth referred to as DIED) and those that survived (henceforth referred to as SUR) within the first 5 days after CLP (Figure 1A). In this (non-sacrificed) group, 20 μl of blood were collected daily for the first 5 days post-CLP to measure selected metabolic and organ injury/dysfunction parameters (i.e. urea, lactate dehydrogenase, alanine transaminase and glucose). Of note, creatinine and cystatin C were not measured in those mice given the severe restriction in the sampled blood volume.

In the CLP-ODam group, mice underwent CLP combined with application of CCl_4_ and cisplatin (see above). Of note, the combination of CCl_4_/cisplatin and CLP was chosen given that it created an environment in which development of organ-injury was additionally promoted via the sepsis-triggered inflammation. Sham surgeries were not performed to reduce the total number of animals.

In the second experiment, early outcome was prospectively predicted via repeated rectal body temperature (BT) measurements using the Fluke 52 Series II thermometer (Fluke, Everett, WA, USA). For example, whenever BT of a given mouse decreased below 28°C (recorded at least at two sequential measurements), the animal was assigned to predicted to die (within the next 24 h) group (henceforth referred to as P-DIE). In the prospective stratification approach, mice were sacrificed in pairs. Specifically, each P-DIE mouse was then sacrificed together with one CLP mouse predicted to survive (henceforth referred to as P-SUR) that was defined by BT ≥ 35°C (Figure 1B). A separate group of healthy control mice (no CLP) was also sacrificed as reference. Blood and organs (kidney, liver, heart and lung) were collected for further analysis.

### Blood sampling

In the first experiment, irrespective of the group, 20 μl of blood were drawn daily for 5 days or until death, including one extra sample at 6 h post-CLP. Blood was collected by puncturing the facial vein (*vena submandibularis*) with a 23-gauge needle as previously described [36]. All samples were collected with a pipette rinsed with ethylenediaminetetraacetic acid (EDTA) (diluted 1:50) and immediately diluted 1:10 in PBS. After centrifugation (1,000×*g*, 5 min, 22°C), 180 μl of plasma was removed and stored at −80°C until further analysis.

In the second experiment, the whole blood was collected at sacrifice from the inferior vena cava with addition of sodium citrate (9:1 ratio vol/vol, blood:citrate). After centrifugation (2,500×*g*, 5 min, 22°C), undiluted plasma was removed and stored at −80°C for further analysis.

### Metabolic and organ injury/function parameters

In the first experiment (28 day follow-up), urea nitrogen (urea), glucose, lactate dehydrogenase (LDH) and alanine transaminase (ALT) were analyzed in plasma samples with Cobas c111 analyzer (Roche, Basel, Switzerland). The lower detection limit for urea was 3 mg/dl, for glucose 1.98 mg/dl, for LDH 10U/l, and for ALT was 2U/l. In the second experiment (BT-based outcome stratification), cystatin C (Biovendor, Karasek, Czech Republic) and troponin I (Life Diagnostics, West Chester, PA, USA) were analyzed in plasma (obtained from undiluted whole blood samples) using ELISA kits according to manufacturer's recommendations. Creatinine was analyzed with Cobas c111 analyzer using the same plasma samples. The lower detection limit for cystatin C was 0.04 ng/ml, for troponin 0.156 ng/ml. In the context of the liver, it must be noted that ALT and LDH are indicators of hepatocyte and general (LDH) tissue injury, thus, they do not *per se* define the level of liver dysfunction (although the latter effect typically develops once a pronounced injury occurs - as in CLP-ODam mice). Given that the parameters we recorded in this study are indicative of either organ injury or (dys)function, we refer to those data by the ‘organ injury/dysfunction’ acronym.

### ATP measurement

In the second experiment (BT-based outcome stratification), the lateral liver lobe, the entire heart and the left kidney were immediately snap-frozen in liquid nitrogen after dissection. Eighty milligrams of each dissected organ was homogenized (RW 1 basic homogenizer, IKA, Wilmington, NC, USA) with three volumes (1:4 wt/vol) of Tris-HCl buffer (20 mM Tris, 135 mM KCl, pH 7.4). Nine hundred microliters of boiling 100 mM Tris/4 mM EDTA buffer (pH 7.75) were added to 100 μl homogenate (liver, kidney, heart). Homogenates (performed as duplicates) were incubated for 2 min at 100°C and then centrifuged (1,000×*g*) for 1 min. The supernatants were transferred to a fresh tube and stored on ice until measurement. ATP content from the supernatant was detected according to the manufacturer's manual (ATP Bioluminescence Assay Kit CLS II, Roche, Switzerland).

### Mitochondrial respiration

In the second experiment (BT-based outcome stratification), respiratory parameters of mitochondria were monitored using a high-resolution respirometer (Oxygraph-2k, Oroboros Instruments, Innsbruck, Austria). Liver/kidney homogenate was incubated in buffer containing 105 mM KCl, 5 mM KH_2_PO_4_, 20 mM Tris-HCl, 0.5 mM EDTA and 5 mg/ml fatty acid-free bovine serum albumin (pH 7.2, 25°C). State 2 respiration was stimulated by addition of 5 mM glutamate/5 mM malate or 10 mM succinate and 1 ng/ml rotenone. Transition to state 3 respiration was induced by addition of adenosine diphosphate (ADP, 1 mM). Respiratory control (RC), characterizing coupling between oxidation and phosphorylation, was determined as the ratio of respiration rates of state 3 to state 2. Mitochondrial respiration in the uncoupled state (total capacity of the respiratory chain) was measured by titration of CCCP in steps of 0.5 μM. Effect of exogenous cytochrome c (2.5 μM) on the respiration rate was used to estimate the permeability of the outer mitochondrial membrane. All reagents were obtained from Sigma-Aldrich (Vienna, Austria).

### Histology and histology scores

In the second experiment (BT-based outcome stratification), histologic slides of organs were stained using hematoxylin-eosin (HE) - and TdT-mediated dUTP-biotin nick end labeling (TUNEL) staining method. All slides were evaluated and scored (blindly) by the pathologist (DG Remick).

### Statistical analysis

The 28-day survival curve was plotted using the Kaplan-Meier method. Accuracy of outcome prediction via BT was analyzed using the receiver operating characteristic (ROC) defined by the area under the curve (AUC) (Figure 1C). The accuracy of the ROC-AUC test is defined as excellent from 0.9 to 1, good from 0.8 to 0.9, fair from 0.7 to 0.8, poor from 0.6 to 0.7, and <0.6, not useful. To ensure maximal data reproducibility, all experiments were conducted in small animal groups (typically 10 to 20 per cycle). All data are presented on the original scale and the level of significance was set at *p* < 0.05.

**Figure 2:**
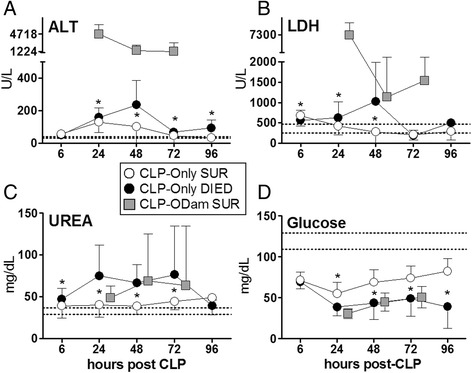
**Retrospective comparison of organ function between dying and surviving septic mice using CLP as reference point.** Plasma levels of **(A)** ALT **(B)** LDH **(C)** urea and **(D)** glucose in mice that died (CLP-Only DIED) or survived (CLP-Only SUR) post-CLP were compared to surviving CLP-ODam mice (CLP-ODam SUR). For **(A)** to **(D)** in CLP-Only: at 6 h, DIED *n* = 45 and SUR *n* ≥ 36; at 24 h, DIED *n* = 37 and SUR *n* = 38; at 48 h, DIED *n* = 13 and SUR *n* ≥ 37; at 72 h, DIED *n* = 7 and SUR *n* ≥ 34; at 96 h, DIED *n* = 3, SUR *n* = 36. In CLP-ODam SUR *n* = 3 at all measured time points (24, 48, 72 h); *p < 0.05 between CLP-Only DIED and CLP-ODam SUR. Dotted lines indicate normal range. Data points shown as mean ± SD.

**Figure 3:**
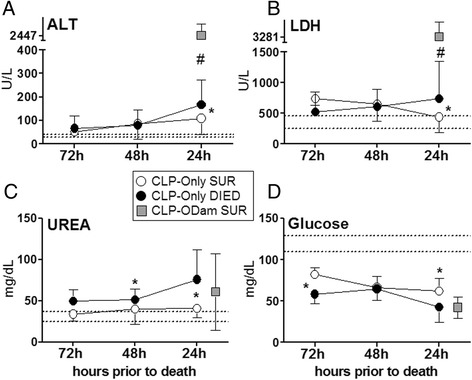
**Retrospective comparison of organ function between dying and surviving septic mice using death as reference point**. Plasma levels of **(A)** ALT, **(B)** LDH, **(C)** urea and **(D**) glucose in mice subjected to CLP were compared between those that either died (CLP-Only DIED) or survived (CLP-Only SUR) and additionally to surviving CLP-ODam mice (CLP-ODam SUR). For (A) to (D) in the CLP-Only group: at 72 h, DIED *n* = 9 and SUR *n* = 35; at 48 h, DIED *n* = 33 and SUR *n* ≥ 30; at 24 h, DIED *n* = 42 and SUR *n* = 40. In the CLP-ODam SUR group, an average value of all combines measurements (i.e. taken at 24, 48 and 72 h post-CLP; *n* = 9) is shown at the 24 h prior death time point; *p < 0.05 between CLP-Only DIED and SUR #*p* < 0.05 between CLP-Only DIED and CLP-ODam SUR. Dotted lines indicate normal range. Data points shown as mean ± SD.

In the first experiment (Figures 2 and 3), a high number (*n* = 83) of enrolled mice (in the CLP-Only group) was required to enable reliable DIE vs. SUR comparison over the 5-day post-CLP period (the DIE subgroup at 72 and 96 h time points was the main limiting factor). Initially, *n* = 10/DIE group at each time point was set to detect a minimal inter-group SUR/DIE difference of approximately 20% to 30% (at *α* = 0.05, *β* = 0.8). The interim data analysis after five CLP runs showed futility of the n = 10 goal for DIE mice at the 96 h time point (due to low late mortality) as well as acceptable data distribution and variability for the pre-96 h time points. Consequently, the study was stopped after reaching *n* DIE = 7 (at 72 h) and n DIE = 3 (at 96 h) and *n* DIE = 9 at 72 h in the inverted comparison approach (Figure 3).

**Figure 4:**
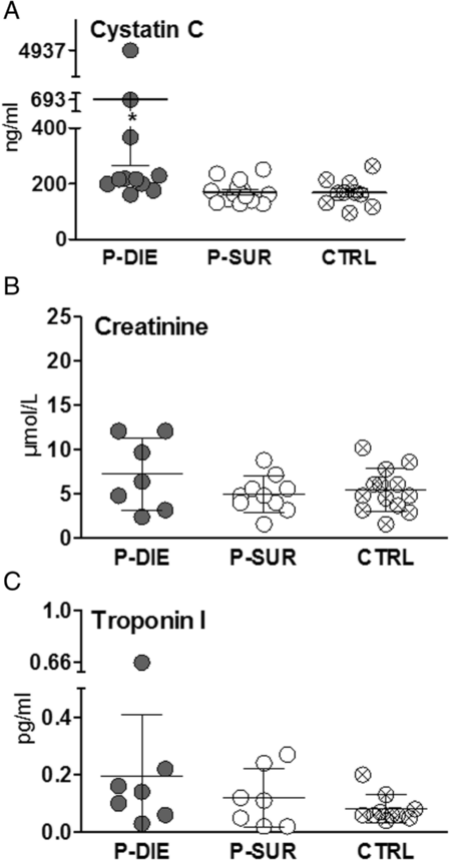
**Comparison of organ function between dying and surviving septic mice using prospective stratification of outcome.** Mice were subjected to CLP, monitored for BT and stratified into either predicted to die (P-DIE) or predicted to survive (P-SUR). Upon identification, P-DIE and P-SUR mice were always sacrificed in pairs additionally compared to the healthy animals (CTRL). **(A)** Cystatin C: P-DIE *n* = 11, P-SUR *n* = 14 and CTRL *n* = 14. **(B)** Creatinine: P-DIE *n* = 7, P-SUR *n* = 10 and CTRL *n* = 13. **C**. Troponin I: P-DIE *n* = 7, P-SUR *n* = 7 and CTRL *n* = 10. *p < 0.05 versus all other groups. Solid horizontal bars indicate mean ± standard error of means (SEM).

**Figure 5:**
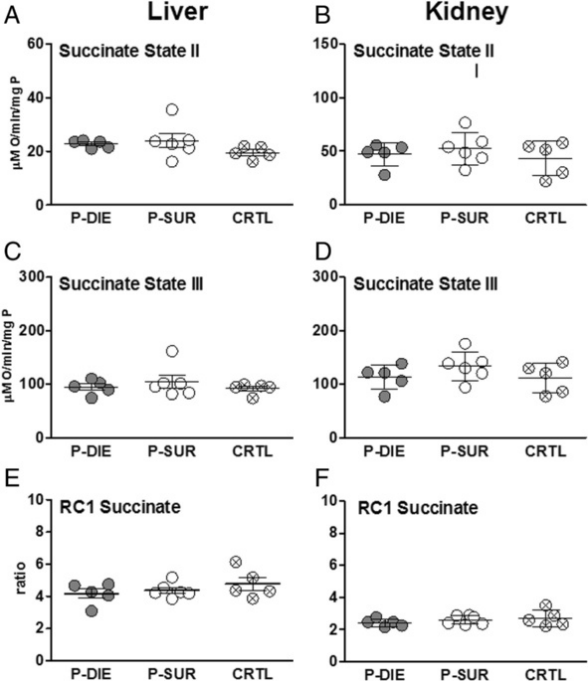
**Comparison** **of mitochondrial function between dying and surviving septic mice using prospective stratification of outcome**. Mice were subjected to CLP, monitored for BT and stratified into either predicted to die (P-DIE) or predicted to survive (P-SUR). Upon identification, P-DIE and P-SUR mice were always sacrificed in pairs and additionally compared to the healthy animals (CTRL). **(A,B**) Succinate state II. **(C,D)** Succinate state III. **(E,F)** Succinate RC. For all parameters: P-DIE *n* = 5, P-SUR *n* = 6 and CTRL *n* = 5. Solid horizontal bars indicate mean ± SEM.

**Figure 6:**
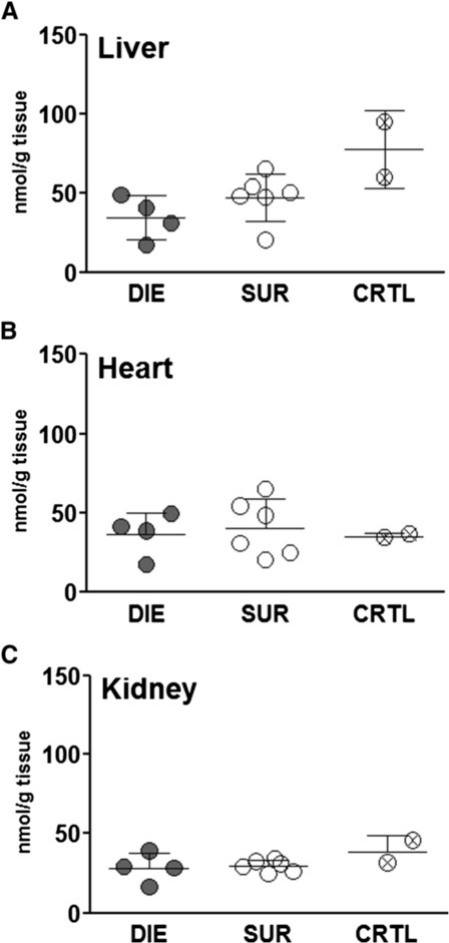
**Comparison of mitochondrial ATP level between dying and surviving septic mice using prospective stratification of outcome.** Mice were subjected to CLP, monitored for BT and stratified into either predicted to die (P-DIE) or predicted to survive (P-SUR). Upon identification, P-DIE and P-SUR mice were always sacrificed in pairs (days 1 to 3 post-CLP) and additionally compared to the healthy animals (CTRL). **(A)** Liver. **(B)** Heart. **(C)** Kidney. For all parameters: P-DIE *n* = 4, P-SUR *n* = 6 and CTRL *n* = 2. Solid horizontal bars indicate mean ± SEM.

In the second experiment, *n* ≥ 10 in the P-DIE and P-SUR groups (i.e. at least ten pairs) was set for comparison of the organ dysfunction score (Table 1) and *n* ≥ 5/group for comparison of other parameters (Figures 4, 5, 6). The *n* inconsistency among individual tables and figures is due to the fact that not all shown parameters could have been measured in each sacrificed mouse and/or due to infrequent errors at sample collection and analysis.

**Table 1:** **Pathology** **score in organs**

**Group**	**Heart**	**Kidney**	**Lung**	**Liver**
CLP-DIED	0	0.5	0.5	0.8
CLP-SUR	0	0.3	0.1	0.1
CONTROL	0	1.0	0	0.8

Scored organs were harvested from CLP-Only mice stratified using the prospective BT-based prediction of outcome and sacrificed on days 1 to 3 post-CLP. P-DIE and P-SUR mice were sacrificed in pairs (*n* = 11/each group) whenever a P-DIE mouse was identified. *N* = 7 in control (healthy individuals). A five-level scoring scale was used: 0 = normal, 1 = minor, 2 = medium, 3 = advanced and 4 = very severe injury.

All organ parameter values were tested for normality before further analysis. Detailed *n* distribution per group is defined in the legend to each figure/table. Subgroup comparisons with at least three groups were made using either one-way ANOVA and Bonferroni for selected pairs as *post hoc* test (parametric data) and Kruskal-Wallis and Dunn's *post hoc* test for selected pairs (non-parametric data). Differences between DIED and SUR at a specific time point were analyzed using unpaired Student's *t*-test (with Welch correction for unequal variances whenever necessary) or Mann-Whitney *U* test for those parameters with non-Gaussian distribution. All statistical analyses and graphics were made with GraphPad (San Diego, CA, USA).

## Results

### Retrospective and prospective comparison of outcome-dependent responses in acute CLP sepsis

The study was divided into two experimental parts. In the first (retrospective) experiment, female OF-1 mice were subjected to CLP, sampled for blood (without sacrifice, see ‘Blood sampling’ section) and observed for 28 days for outcome. Forty-eight percent of those animals were alive at the end of the observation period (Figure 1A) and all data obtained between days 1 and 5 post-CLP were retrospectively compared based on the actual outcome.

In the second (prospective) experiment, we intended to prospectively predict the outcome of CLP-induced sepsis based on specific BT cutoff values given that in septic mice, a strong decrease of BT is among the distinct signs of upcoming death. Therefore, a group of female OF-1 mice was subjected to CLP, monitored BT for first 5 days (post-CLP) and divided into sub-groups with predicted outcome; the prospective identification approach is presented in Figure 1B schematic. We had established an excellent accuracy of BT to predict outcome (AUC = 0.93) by ROC analysis using our previously compiled internal BT measurement database: identification of P-DIE mice (BT ≤ 28°C) had 94% specificity, while identification of P-SUR mice (BT ≥ 35°C; at least for the next 24 h) displayed 100% specificity (Figure 1C).

In this study, an average of 23% of mice per day did not meet the low/high temperature cutoffs for the outcome prediction. The exact percentages of animals >28°C and <35°C for each sampling time point post-CLP were: 50% at 6 h, 27% at 24 h, 12% at 48 h, 18% at 72 h and 8% at 96 h.

### CLP as reference point: temporal profiles of organ injury/dysfunction in dying and surviving septic mice

As polymicrobial sepsis unfolds, homeostasis of vital organs becomes deregulated. By using our serial low-volume sampling approach (first experiment) with CLP as the reference point, we followed temporal fluctuations of circulating organ function/injury parameters in DIED and SUR septic (CLP-Only) mice juxtaposing them with the readouts from non-lethal CLP-ODam mice.

In the CLP-Only group, a consistent deterioration in all parameters was recorded in DIED versus SUR mice. At 48 h post-CLP (the most pronounced DIED/SUR separation), circulating ALT in DIED was 2.3-fold higher (vs. SUR) (235 vs. 102 U/l), LDH was 3.7-fold higher (1,028 vs. 276 U/l) and urea was 1.7-fold higher (67 vs. 39 mg/dl), while blood glucose decreased by approximately 30% (44 vs. 69 mg/dl) (Figure 2).

We wanted to gain more insight into the biological relevance of the recorded differences upon acute septic outcomes, therefore, we compared temporally measured values (at 24, 48 and 72 h post-CLP) obtained from CLP-ODam survivors to the corresponding measurements obtained in DIED and SUR mice from the CLP-Only cohort. The deterioration of readouts in surviving CLP-ODam mice was always greater than in SUR and either equaled or further exceeded changes recorded in DIED CLP-Only. In CLP-ODam mice, ALT was 5- to 30-fold higher (up to 4,717 U/l), LDH 8- to 11-fold higher (up to 7,300 U/l) while urea and glucose were approximately equal to the values measured at 24 to 72 h time points in DIED mice of the CLP-Only group (Figure 2A-D).

### Death as reference point: pre-lethal evolution of organ injury/dysfunction in acute CLP

Given that organ dysfunction/injury in DIED mice was more pronounced at many but not all time points compared to SUR, we next aimed to characterize the dynamics of progressive pre-lethal organ impairment in acute CLP. Therefore, we used the time of impending death as the reference point to inversely plot changes in the recorded parameters between dying and surviving CLP-Only mice (and also to compare them to CLP-ODam survivors).

Within 48 h of death, apart from an approximately 30% increase in urea (51 vs. 40 mg/dl), none of the parameters recorded in the DIED group was higher than in corresponding SUR CLP-Only mice. However, a significantly higher deterioration of all parameters was recorded within 24 h of death: ALT increased by 54% (167 vs. 107 U/l), LDH by 68% (739 vs. 439 U/l), urea by 86% (76 vs. 40 U/l), while glucose decreased by 30% (62 vs. 43 mg/dl). Next, the last pre-lethal (i.e. at 24 h time point) measurements in DIED CLP-Only were compared to the averaged value (i.e. using the combined 24, 48 and 72 h post-CLP measurements) of each respective parameter measured in CLP-ODam survivors. The comparison demonstrated that in CLP-ODam survivors, ALT was 15-fold higher (167 vs. 2,447 U/l), LDH was 5-fold higher (739 vs. 3,281 U/l) while urea and glucose were approximately similar to CLP-Only mice that died (Figure 3A-D).

By using the ROC analysis, we also estimated the capacity of the last pre-death measurements (i.e. within 24 h of death) to predict early CLP outcome (Table 2). Only the urea (and blood urea nitrogen (BUN); Additional file 1: Figure S1) demonstrated a good (AUC = 0.85) separation between DIED and SUR datasets: 40% of CLP mice would have been identified as predicted to die without inclusion of false positive SUR mice.

**Table 2:** **Capacity to predict outcome in acute CLP sepsis**

**Parameter**	**AUC**	**Cutoff**	**Specificity (%)**	**Sensitivity (%)**
Urea	0.85	77 mg/dl	100	40
Glucose	0.78	30 U/l	100	25
LDH	0.68	1,320 U/l	100	17
ALT	0.68	365 U/l	100	7

CLP-Only mice were never sacrificed but sampled daily for blood between days 1 to 5 post-CLP and followed for 28 days. For ROC analysis, parameter values recorded within 24 h of death (occurring anytime between days 1 and 5 post-CLP) and from mice that lived until day 28 post-CLP were used. Specificity was set to 100% to identify only mice predicted to die without committing the type I error (i.e. an erroneous inclusion of P-SUR animals). ROC calculation was based on: *n* = 42 for mice that died within days 1 to 5 and *n* = 40 for mice that survived until day 28 post-CLP.

### Outcome-dependent profiling of kidney and cardiac muscle tissue injury in acute CLP based on the BT prediction

In the next step, we aimed to characterize the magnitude of kidney dysfunction and cardiac muscle tissue injury in fully symptomatic mice dying of sepsis. We assessed creatinine and cystatin C as markers for renal glomerular filtration rate and troponin I as marker for cardiac muscle tissue injury. In CLP-Only mice predicted to die, circulating cystatin C rose by approximately 1.6-fold (692 vs. 170 ng/dl) (*p* < 0.05) while creatinine was not higher than SUR (and control) animals (Figure 4A,B). Troponin I levels did not differ among P-DIE, P-SUR (CLP-Only) and healthy control groups (Figure 4C). To comply with the three Rs principle, a comparison to CLP-ODam survivors was not performed (explained in the ‘CCl_4_/cisplatin model’ section).

### Outcome-dependent profiling of renal and hepatic mitochondrial respiratory activity in acute CLP based on the BT prediction

Pronounced organ failure is typically preceded by more subtle metabolic changes at the cellular level. To investigate the cellular energy homeostasis in CLP-induced sepsis, we compared the outcome-dependent activity of the mitochondrial respiratory electron transport chain in septic mice. Specifically, we assessed state 2/state 3 respirations and RC in the presence of succinate as well as ATP level in organs collected from P-DIE and P-SUR CLP-Only mice (and healthy control).

In the liver and kidney, succinate state 2 and 3 did not differentiate between P-DIE, P-SUR and control mice (Figure 5A,B). Similarly, RC was virtually identical among groups, irrespective of the outcome and organ (Figure 5C).

Next, a direct measurement of ATP was performed in the liver, heart and kidney of CLP-Only mice predicted to either die or survive (and healthy control). ATP concentration was virtually identical among all three groups in each examined organ (Figure 6A-C).

### Acute CLP did not induce prominent pathomorphologic changes in organs

**Figure 7:**
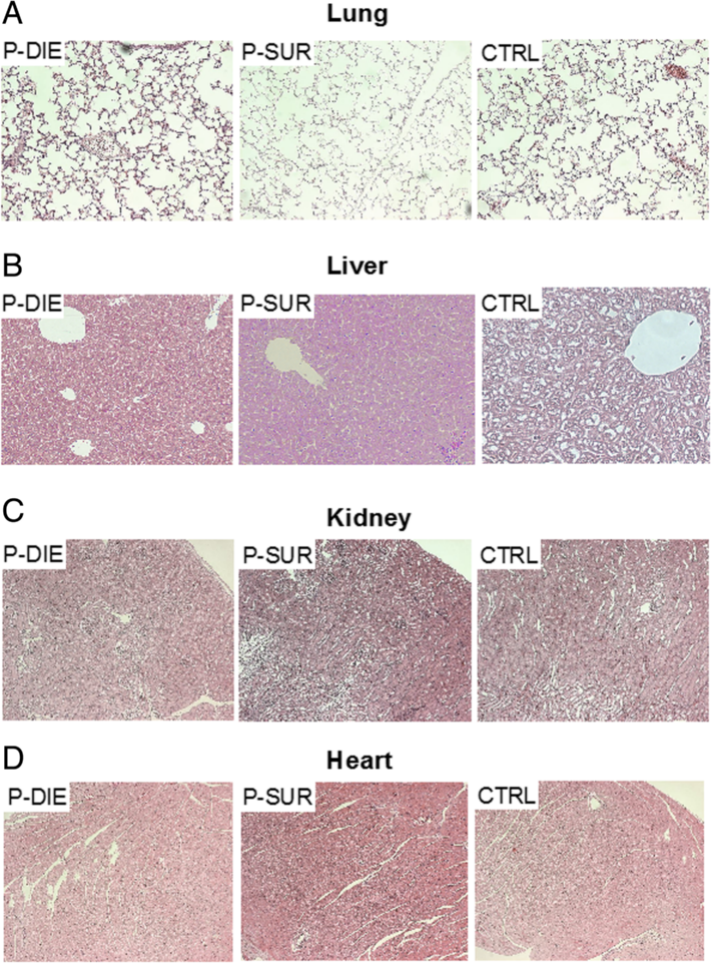
**Comparison of pathomorphologic changes mitochondrial function between dying and surviving septic mice using prospective stratification of outcome.** Mice were subjected to CLP, monitored for BT and stratified into either predicted to die (P-DIE) or predicted to survive (P-SUR). Upon identification, P-DIE and P-SUR mice were always sacrificed in pairs (days 1 to 3 post-CLP) and additionally compared to the healthy animals (CTRL). HE-staining of **(A)** lung, **(B)** liver, **(C)** kidney and **(D)** heart was performed. P-DIE and P-SUR *n* = 11 and CTRL *n* = 7. Representative photographs are shown (original magnification ×10).

In the final investigative step, organ samples collected from CLP-Only mice were subjected to histologic evaluation for detection of pathomorphologic signs of organ damage. Based on the comparison of combined pathology scores, there were no distinct signs of organ injury in neither of the examined groups (Table 1). Representative micrographs of the examined organs are shown in Figure 7. Similarly, the TUNEL assay revealed only trivial changes: only isolated apoptotic cells in the lungs and liver of septic mice were observed (Additional file 2: Figure S2).

## Discussion

Overall, given the absence of cellular respiratory/energy disturbances and histopathological damage as well as the relatively mild exacerbation of organ injury/dysfunction (as measured by selected circulating parameters), this study strongly cautions against reflexive interpretation of organ deregulation as the main culprit of early deaths in the young outbred CLP female mice. The factual contribution of the recorded organ injury/dysfunction to early CLP mortality strongly depends on the pathophysiological and immuno-inflammatory context in which those detrimental changes occurred.

To investigate early organ dysfunction/injury in sepsis, this study integrated a number of innovative design elements. We simulated the routine daily blood monitoring and treatment of hospitalized septic patients (by using broad-range antimicrobial treatment, fluid resuscitation and analgesics). The CLP severity level was carefully set to recapitulate the mortality of patients suffering from abdominal sepsis (30% to 50%) [37]. This is important given that both the untreated systemic infection as well as models with excessive severity may strongly skew the model towards the more pronounced organ damage and artificially pre-program the outcome [1]. Furthermore, we used two stratification approaches: an outcome-dependent retrospective [24,25] and BT-based prospective one [38] - both allow a comparison between confirmed dying and surviving CLP mice (see the stratification section in ‘Methods’) instead of the typical approach that compares septic and control (healthy) mice.

In the retrospective stratification approach, we first compared 5-day profiles using CLP as the reference point. While a statistically significant separation of four circulating parameters was recorded between DIED and SUR mice, this difference was not consistent at all time points. For example, ALT and LDH separation was greatest at 48 h post-CLP, while urea increase and glucose decrease began early and remained constantly separated. The delay observed in ALT and LDH dynamics is consistent with our recent post-traumatic sepsis study, which demonstrated the greatest difference in a combined organ score between DIE and SUR mice at 48 h post-CLP [24]. In two rodent studies, liver dysfunction in abdominal sepsis was described as an early event and signs of acute hepatic failure coincided with early deaths [34,39]. Specifically, Recknagel et al. using a rat model of fecal peritonitis demonstrated that sepsis-related hepatic malfunction (already present at 6 h post-challenge) was more severe in animals predicted to die [39]. Thanks to the low-volume sampling technique [36], we also analyzed data by using the day of death as the reference point. This approach enabled us to profile dynamic parameter changes towards the approaching death - similar to deteriorating ICU septic patients in whom the onset of sepsis is typically unknown. It was striking to observe that the separation of DIED vs. SUR mice became clear only within the last 24 h (prior to death); no signs of differential organ deterioration were apparent at earlier time points.

Yet, the above findings did not resolve the main question: i.e. to what extent the recorded level of exacerbation of organ injury/dysfunction was responsible for the acute mortality. We attempted to address it, at least partly, by comparing CLP-Only to mild CLP with administered CCl_4_ and cisplatin to provoke an added exacerbation of organ injury/dysfunction (see respective sections in ‘Methods’). Remarkably, although organ injury and glycemic deregulation in septic ODam mice either approximately equaled (based on urea and glucose) or dramatically exceeded (based on ALT and LDH) the ones recorded in DIED CLP-Only mice, they did not lead to death. It must be noted that comparison of those two experimental setups (i.e. CLP-Only and CLP-ODam) is not ideal considering the dissimilar release mechanism of the measured biomarkers: pronounced hepatocyte (cirrhotic) necrosis caused ALT/LDH secretion in CLP-ODam mice [26], while their release in sepsis (i.e. CLP-Only) can be attributed to circulatory disturbances (e.g. decreased arterial hepatic blood flow) rather than necrosis/apoptosis [1]. Nevertheless, two assumptions can be risked based on the above data. First, the pathophysiological origin of the damage (e.g. presence of comorbidity) can dramatically alter the circulation dynamics of a given organ injury/function parameter(s) in the CLP model (and sepsis in general). Second, even when the elevation of those parameters is very high, it should not be automatically judged as indication of a lethal organ failure and the main cause of early death in CLP sepsis. A recent study by Peng et al. [40] demonstrated a similar ‘mismatch’ phenomenon: an improved survival in antibiotic-treated CLP (24 to 28 weeks old) rats coincided with a much higher severity of AKI (defined by RIFLE score) compared to rats with milder AKI but an exacerbated mortality. In related examples, a trichostatin A-dependent improvement of CLP-induced hepatic injury in C57BL/6 mice did not increase their survival, and a similar lack of survival advantage was true in the CORTICUS trial septic patients in whom hydrocortisone treatment strongly attenuated organ injury [41].

To gain a deeper insight into the severity of organ dysfunction/injury in our medium-severity CLP sepsis model, we next employed a prospective BT-based stratification that allowed autopsy and examination of tissue histology and mitochondrial energy metabolism in organs. In acute sepsis, factors like pulmonary damage, reduction of cardiac output and impaired myocardial contractibility may lead to shortage of oxygen and consequently to a failure of mitochondrial respiration and/or tissue energy status (tissue ATP level) in major organs [42,43]. Yet, the role of mitochondrial dysfunction in the septic organ failure and subsequent death is unclear [43,44]. In our study, we did not observe any disturbances in mitochondrial respiration and energy status in the examined organs (i.e. liver, heart, kidney); the sick mice, regardless whether P-DIE or P-SUR, were undistinguishable from the healthy subjects. The unaltered ATP level in the hearts of septic mice is in line with their stable level of circulating troponin I - an established marker of myocardial tissue injury and prognosticator of mortality in severe sepsis patients [45,46]. The histological assessment of the organs demonstrated only random alterations (accompanied by only sporadic apoptosis) further reinforcing the perception that the recorded exacerbation in circulating markers in P-DIE (vs. P-SUR), although statistically significant, was not immediately indicative of a lethal organ failure in the young OF-1 mice. Of note, the autopsied organs were harvested from fully symptomatic animals within the hours of their demise (i.e. by prospective BT-based stratification), thus, maximizing the probability of detecting signs of structural damage in the examined tissues.

The issue of kidney (dys)function in sepsis models draws special attention. In sepsis, renal blood flow and microcirculation become impaired leading to an acute tubular injury, cell death and kidney shutdown [47]. In the ICU patients with sepsis and/or MODS, the presence of AKI strongly and independently correlates with exacerbated mortality [48]. In rodents, the CLP model has been frequently shown to produce AKI demonstrable both on functional and structural level [49-53]. In contrast to those studies, we did not observe any kidney injury in the young OF-1 females based on circulating creatinine and histologic examinations. Yet, compared to survivors, pre-lethal measurements in mice dying within the next 24 h (identified by pro-and retrospective stratification) demonstrated a consistent increase in two other markers: blood urea (and BUN) and cystatin C. Notably, these findings are virtually identical with the most recent study published by Craciun et al. [54], who compared kidney dysfunction in 8-week-old female outbred ICR mice stratified into dying and surviving cohorts (based on circulating IL-6). For example, BUN/urea from the cited study showed a similar predictive potential for early CLP deaths to the one we recorded (AUC = 0.85; Additional file 1: Figure S1 and Table 2); in both studies, this phenomenon was true despite negative creatinine readouts. This collectively suggests that while a certain level of renal dysfunction indeed develops, those changes do not appear as the key causal factor in early CLP deaths. Furthermore, and in parallel to clinical findings [55,56], it becomes obvious that creatinine alone does not qualify as an optimal marker for detection of AKI in the mouse, while cystatin C appears to be useful [53,56,57]. Finally, it is also suggestive that the mouse strain heavily influences the magnitude, severity and the factual contribution of AKI to outcomes in experimental sepsis. Best to our knowledge, only inbred [49,51,53,58,59] but not outbred mouse strains were shown to display patient-like AKI alterations. This is consistent with the occurrence (or absence) of CLP-induced acute lung injury (ALI): we recently showed that outbred ICR mice do not develop significant lung injury [16], while others produced ALI in inbred BALB/c and C57/BL/6 strains [58,60].

The most important shortcoming of our study is that we did not assess all building blocks of clinically defined MODS (e.g. acute respiratory distress, hypotension, diminished cardiac output). Due to obvious technical limitations, we were able to focus only on selected aspects of organ injury/dysfunction and supplementary studies are necessary to expand onto those omitted elements. In consequence, our preclinical study is not definite and, furthermore, it is not our intention to imply that organ dysfunction developing in septic patients should be discounted as the main contributor to their mortality. The main strength of this study lies elsewhere: it strongly cautions that findings from numerous preclinical experiments, due to their varying design, are prone to severe misinterpretations [61]. In other words, we believe that preclinical studies conducted in young and healthy mice tend to over interpret statistically significant, but relatively mild and not instantly life-threatening changes in organ function/injury as: (1) the leading cause of septic death, and/or (2) the key life-saving mechanism of action behind beneficial effects of tested therapeutics.

## Conclusions

It is clear that the dynamics of organ dysfunction/injury and its response to treatments profoundly differ between young mice without underlying comorbidities and their aged and disease-burdened counterparts as well as between the inbred and outbred lineages. The past year has brought about a heated discussion regarding the true utility of mouse critical care disease models [62,63]. Among other things, this discussion has stressed that the mistrust of data from sub-optimally matched/designed experiments can also easily encompass relevant preclinical studies given that recognition of the various design flaws is not easy and immediate [61,64,65]. Therefore, we believe that the degree of the interpretational skepticism applied to the mouse organ dysfunction-related experiments should be proportional to the distance separating their study design quality from the clinical reality.

## Additional files

Additional file 1: Figure S1. Comparison of BUN elevation between dying and surviving CLP mice. **(A)** Plotted dots represent blood urea nitrogen (BUN) values taken from mice within 24 h of death (occurring anytime between days 1 and 4 post-CLP; right scatter) and from mice that lived until day 28 post-CLP (left scatter). Surviving *n* = 40; dying *n* = 42. **(B)** Predictive capacity of BUN for outcome by ROC curve assessed by measurements taken within 24 h of death in the acute phase of CLP sepsis (days 1 to 4 post-CLP). Each dot represents a single mouse. Surviving *n* = 40; dying *n* = 42. Solid horizontal lines represent mean ± SD. The two dotted horizontal lines represent 95% CI. **p* < 0.05. Additional file 2: Figure S2. Comparison of apoptosis in different organs between dying and surviving septic mice using prospective stratification of outcome. Mice were subjected to CLP, monitored for BT and stratified into either predicted to die (P-DIE) or predicted to survive (P-SUR). Upon identification, P-DIE and P-SUR mice were always sacrificed in pairs (days 1 to 3 post-CLP) and additionally compared to the healthy animals (CTRL). TUNEL-staining of **(A)** lung, **(B)** liver, **(C)** kidney and **(D)** heart was performed. P-DIE and P-SUR *n* = 11 and CTRL *n* = 7. Representative photographs are shown (original magnification ×10). Arrow in (A) indicates an infrequent apoptotic cell.

## References

[1] Iskander KN, Osuchowski MF, Stearns-Kurosawa DJ, Kurosawa S, Stepien D, Valentine C, Remick DG (2013). Sepsis: multiple abnormalities, heterogeneous responses, and evolving understanding. Physiol Rev.

[2] O'Brien JM, Ali NA, Abraham E (2008). Year in review 2007: critical care - multiple organ failure and sepsis. Crit Care.

[3] Vincent JL, Sakr Y, Sprung CL, Ranieri VM, Reinhart K, Gerlach H, Moreno R, Carlet J, Le G, Payen D (2006). Sepsis in European intensive care units: results of the SOAP study. Crit Care Med.

[4] Bernard GR, Vincent JL, Laterre PF, LaRosa SP, Dhainaut JF, Lopez-Rodriguez A, Steingrub JS, Garber GE, Helterbrand JD, Ely EW, Fisher CJ (2001). Efficacy and safety of recombinant human activated protein C for severe sepsis. N Engl J Med.

[5] Vincent JL, Nelson DR, Williams MD (2011). Is worsening multiple organ failure the cause of death in patients with severe sepsis?. Crit Care Med.

[6] de Montmollin E, Annane D (2011). Year in review 2010: critical care - multiple organ dysfunction and sepsis. Crit Care.

[7] Waydhas C, Nast-Kolb D, Jochum M, Trupka A, Lenk S, Fritz H, Duswald KH, Schweiberer L (1992). Inflammatory mediators, infection, sepsis, and multiple organ failure after severe trauma. Arch Surg.

[8] Regel G, Sturm JA, Pape HC, Gratz KF, Tscherne H (1991). Multiple organ failure. Reflection of generalized cell damage of all organs following severe trauma. Unfallchirurg.

[9] Maier B, Lefering R, Lehnert M, Laurer HL, Steudel WI, Neugebauer EA, Marzi I (2007). Early versus late onset of multiple organ failure is associated with differing patterns of plasma cytokine biomarker expression and outcome after severe trauma. Shock.

[10] Regel G, Grotz M, Weltner T, Sturm JA, Tscherne H (1996). Pattern of organ failure following severe trauma. World J Surg.

[11] Osterbur K, Mann FA, Kuroki K, DeClue A (2014). Multiple organ dysfunction syndrome in humans and animals. J Vet Intern Med.

[12] Proulx F, Fayon M, Farrell CA, Lacroix J, Gauthier M (1996). Epidemiology of sepsis and multiple organ dysfunction syndrome in children. Chest.

[13] Hotchkiss RS, Karl IE (2003). The pathophysiology and treatment of sepsis. N Engl J Med.

[14] Boomer JS, To K, Chang KC, Takasu O, Osborne DF, Walton AH, Bricker TL, Jarman SD, Kreisel D, Krupnick AS, Srivastava A, Swanson PE, Green JM, Hotchkiss RS (2011). Immunosuppression in patients who die of sepsis and multiple organ failure. JAMA.

[15] van Griensven M, Kuzu M, Breddin M, Bottcher F, Krettek C, Pape HC, Tschernig T (2002). Polymicrobial sepsis induces organ changes due to granulocyte adhesion in a murine two hit model of trauma. Exp Toxicol Pathol.

[16] Iskander KN, Craciun FL, Stepien DM, Duffy ER, Kim J, Moitra R, Vaickus LJ, Osuchowski MF, Remick DG (2013). Cecal ligation and puncture-induced murine sepsis does not cause lung injury. Crit Care Med.

[17] Nemzek JA, Hugunin KM, Opp MR (2008). Modeling sepsis in the laboratory: merging sound science with animal well-being. Comp Med.

[18] Nemzek JA, Xiao HY, Minard AE, Bolgos GL, Remick DG (2004). Humane endpoints in shock research. Shock.

[19] Wichterman KA, Baue AE, Chaudry IH (1980). Sepsis and septic shock - a review of laboratory models and a proposal. J Surg Res.

[20] Raeven P, Feichtinger GA, Weixelbaumer KM, Atzenhofer S, Redl H, Van GM, Bahrami S, Osuchowski MF (2012). Compartment-specific expression of plasminogen activator inhibitor-1 correlates with severity/outcome of murine polymicrobial sepsis. Thromb Res.

[21] Dejager L, Pinheiro I, Dejonckheere E, Libert C (2011). Cecal ligation and puncture: the gold standard model for polymicrobial sepsis?. Trends Microbiol.

[22] Torgersen C, Moser P, Luckner G, Mayr V, Jochberger S, Hasibeder WR, Dunser MW (2009). Macroscopic postmortem findings in 235 surgical intensive care patients with sepsis. Anesth Analg.

[23] Osuchowski MF, Craciun F, Weixelbaumer KM, Duffy ER, Remick DG (2012). Sepsis chronically in MARS: systemic cytokine responses are always mixed regardless of the outcome, magnitude, or phase of sepsis. J Immunol.

[24] Drechsler S, Weixelbaumer K, Raeven P, Jafarmadar M, Khadem A, Van GM, Bahrami S, Osuchowski MF (2012). Relationship between age/gender-induced survival changes and the magnitude of inflammatory activation and organ dysfunction in post-traumatic sepsis. PLoS One.

[25] Osuchowski MF, Craciun FL, Schuller E, Sima C, Gyurko R, Remick DG (2010). Untreated type 1 diabetes increases sepsis-induced mortality without inducing a prelethal cytokine response. Shock.

[26] Singh AP, Junemann A, Muthuraman A, Jaggi AS, Singh N, Grover K, Dhawan R (2012). Animal models of acute renal failure. Pharmacol Rep.

[27] Weber LW, Boll M, Stampfl A (2003). Hepatotoxicity and mechanism of action of haloalkanes: carbon tetrachloride as a toxicological model. Crit Rev Toxicol.

[28] Miller RP, Tadagavadi RK, Ramesh G, Reeves WB (2010). Mechanisms of cisplatin nephrotoxicity. Toxins (Basel).

[29] Marques TG, Chaib E, da Fonseca JH, Lourenco AC, Silva FD, Ribeiro MA, Galvao FH, D'Albuquerque LA (2012). Review of experimental models for inducing hepatic cirrhosis by bile duct ligation and carbon tetrachloride injection. Acta Cir Bras.

[30] Ma JQ, Ding J, Zhang L, Liu CM (2014). Ursolic acid protects mouse liver against CCl_4_-induced oxidative stress and inflammation by the MAPK/NF-kappaB pathway. Environ Toxicol Pharmacol.

[31] Daugaard G (1990). Cisplatin nephrotoxicity: experimental and clinical studies. Dan Med Bull.

[32] Oh GS, Kim HJ, Choi JH, Shen A, Choe SK, Karna A, Lee SH, Jo HJ, Yang SH, Kwak TH, Lee CH, Park R, So HS (2014). Pharmacological activation of NQO1 increases NAD^+^ levels and attenuates cisplatin-mediated acute kidney injury in mice. Kidney Int.

[33] Yan J, Li S, Li S (2014) The role of the liver in sepsis. Int Rev Immunol 33:498-510 10.3109/08830185.2014.889129 PMC4160418 24611785

[34] Zhang L, Wan J, Jiang R, Wang W, Deng H, Shen Y, Zheng W, Wang Y (2009). Protective effects of trichostatin A on liver injury in septic mice. Hepatol Res.

[35] Kilkenny C, Browne WJ, Cuthill IC, Emerson M, Altman DG (2010). Improving bioscience research reporting: the ARRIVE guidelines for reporting animal research. PLoS Biol.

[36] Weixelbaumer KM, Raeven P, Redl H, Van GM, Bahrami S, Osuchowski MF (2010). Repetitive low-volume blood sampling method as a feasible monitoring tool in a mouse model of sepsis. Shock.

[37] Volakli E, Spies C, Michalopoulos A, Groeneveld AB, Sakr Y, Vincent JL (2010). Infections of respiratory or abdominal origin in ICU patients: what are the differences?. Crit Care.

[38] Raeven P, Salibasic A, Drechsler S, Weixelbaumer KM, Jafarmadar M, Van GM, Bahrami S, Osuchowski MF (2013). A non-lethal traumatic/hemorrhagic insult strongly modulates the compartment-specific PAI-1 response in the subsequent polymicrobial sepsis. PLoS One.

[39] Recknagel P, Gonnert FA, Westermann M, Lambeck S, Lupp A, Rudiger A, Dyson A, Carre JE, Kortgen A, Krafft C, Popp J, Sponholz C, Fuhrmann V, Hilger I, Claus RA, Riedemann NC, Wetzker R, Singer M, Trauner M, Bauer M (2012). Liver dysfunction and phosphatidylinositol-3-kinase signalling in early sepsis: experimental studies in rodent models of peritonitis. PLoS Med.

[40] Peng ZY, Wang HZ, Srisawat N, Wen X, Rimmele T, Bishop J, Singbartl K, Murugan R, Kellum JA (2012). Bactericidal antibiotics temporarily increase inflammation and worsen acute kidney injury in experimental sepsis. Crit Care Med.

[41] Moreno R, Sprung CL, Annane D, Chevret S, Briegel J, Keh D, Singer M, Weiss YG, Payen D, Cuthbertson BH, Vincent JL (2011). Time course of organ failure in patients with septic shock treated with hydrocortisone: results of the Corticus study. Intensive Care Med.

[42] Garrabou G, Moren C, Lopez S, Tobias E, Cardellach F, Miro O, Casademont J (2012). The effects of sepsis on mitochondria. J Infect Dis.

[43] Galley HF (2011). Oxidative stress and mitochondrial dysfunction in sepsis. Br J Anaesth.

[44] Ruggieri AJ, Levy RJ, Deutschman CS (2010). Mitochondrial dysfunction and resuscitation in sepsis. Crit Care Clin.

[45] John J, Woodward DB, Wang Y, Yan SB, Fisher D, Kinasewitz GT, Heiselman D (2010). Troponin-I as a prognosticator of mortality in severe sepsis patients. J Crit Care.

[46] Mehta NJ, Khan IA, Gupta V, Jani K, Gowda RM, Smith PR (2004). Cardiac troponin I predicts myocardial dysfunction and adverse outcome in septic shock. Int J Cardiol.

[47] Dirkes S (2013). Sepsis and inflammation: impact on acute kidney injury. Nephrol Nurs J.

[48] Levy EM, Viscoli CM, Horwitz RI (1996). The effect of acute renal failure on mortality. A cohort analysis. JAMA.

[49] Wang Z, Holthoff JH, Seely KA, Pathak E, Spencer HJ, Gokden N, Mayeux PR (2012). Development of oxidative stress in the peritubular capillary microenvironment mediates sepsis-induced renal microcirculatory failure and acute kidney injury. Am J Pathol.

[50] Seely KA, Holthoff JH, Burns ST, Wang Z, Thakali KM, Gokden N, Rhee SW, Mayeux PR (2011). Hemodynamic changes in the kidney in a pediatric rat model of sepsis-induced acute kidney injury. Am J Physiol Renal Physiol.

[51] Liu L, Li Y, Hu Z, Su J, Huo Y, Tan B, Wang X, Liu Y (2012). Small interfering RNA targeting Toll-like receptor 9 protects mice against polymicrobial septic acute kidney injury. Nephron Exp Nephrol.

[52] Liu J, bdel-Razek O, Liu Z, Hu F, Zhou Q, Cooney RN, Wang G (2014) Role of surfactant proteins A and D in sepsis-induced acute kidney injury. Shock 43:31-38 10.1097/SHK.0000000000000270 PMC4269566 25255378

[53] Holthoff JH, Wang Z, Patil NK, Gokden N, Mayeux PR (2013). Rolipram improves renal perfusion and function during sepsis in the mouse. J Pharmacol Exp Ther.

[54] Craciun FL, Iskander KN, Chiswick EL, Stepien DM, Henderson JM, Remick DG (2014). Early murine polymicrobial sepsis predominantly causes renal injury. Shock.

[55] Murray PT, Mehta RL, Shaw A, Ronco C, Endre Z, Kellum JA, Chawla LS, Cruz D, Ince C, Okusa MD (2014). Potential use of biomarkers in acute kidney injury: report and summary of recommendations from the 10th Acute Dialysis Quality Initiative consensus conference. Kidney Int.

[56] Chawla LS, Kellum JA (2012). Acute kidney injury in 2011: biomarkers are transforming our understanding of AKI. Nat Rev Nephrol.

[57] Leelahavanichkul A, Souza AC, Street JM, Hsu V, Tsuji T, Doi K, Li L, Hu X, Zhou H, Kumar P, Schnermann J, Star RA, Yuen PS (2014). Comparison of serum creatinine and serum cystatin C as biomarkers to detect sepsis-induced acute kidney injury and to predict mortality in CD-1 mice. Am J Physiol Renal Physiol.

[58] Bhargava R, Altmann CJ, Ndres-Hernando A, Webb RG, Okamura K, Yang Y, Falk S, Schmidt EP, Faubel S (2013). Acute lung injury and acute kidney injury are established by four hours in experimental sepsis and are improved with pre, but not post, sepsis administration of TNF-alpha antibodies. PLoS One.

[59] Lygizos MI, Yang Y, Altmann CJ, Okamura K, Hernando AA, Perez MJ, Smith LP, Koyanagi DE, Gandjeva A, Bhargava R, Tuder RM, Faubel S, Schmidt EP (2013). Heparanase mediates renal dysfunction during early sepsis in mice. Physiol Rep.

[60] Chao MC, Garcia CS, de Oliveira MB, Santos RS, Lucas IH, Silva PL, Vieira-Abreu A, de Castro-Faria-Neto HC, Parra-Cuentas ER, Capelozzi VL, Pelosi P, Rocco PR (2010). Degree of endothelium injury promotes fibroelastogenesis in experimental acute lung injury. Respir Physiol Neurobiol.

[61] Osuchowski MF, Remick DG, Lederer JA, Lang CH, Aasen AO, Aibiki M, Azevedo LC, Bahrami S, Boros M, Cooney R, Cuzzocrea S, Jiang Y, Junger WG, Hirasawa H, Hotchkiss RS, Li XA, Radermacher P, Redl H, Salomao R, Soebandrio A, Thiemermann C, Vincent JL, Ward P, Yao YM, Yu HP, Zingarelli B, Chaudry IH (2014). Abandon the mouse research ship? Not just yet!. Shock.

[62] Seok J, Warren HS, Cuenca AG, Mindrinos MN, Baker HV, Xu W, Richards DR, Donald-Smith GP, Gao H, Hennessy L, Finnerty CC, Lopez CM, Honari S, Moore EE, Minei JP, Cuschieri J, Bankey PE, Johnson JL, Sperry J, Nathens AB, Billiar TR, West MA, Jeschke MG, Klein MB, Gamelli RL, Gibran NS, Brownstein BH, Miller-Graziano C, Calvano SE, Mason PH, Cobb JP, Rahme LG, Lowry SF, Maier RV, Moldawer LL, Herndon DN, Davis RW, Xiao W, Tompkins RG (2013). Genomic responses in mouse models poorly mimic human inflammatory diseases. Proc Natl Acad Sci U S A.

[63] Takao K and Miyakawa T (2014) Genomic responses in mouse models greatly mimic human inflammatory diseases. Proc Natl Acad Sci U S A 112:1167-1172. 10.1073/pnas.1401965111 PMC4313832 25092317

[64] Fink MP (2014). Animal models of sepsis. Virulence.

[65] Gentile LF, Nacionales DC, Lopez MC, Vanzant E, Cuenca A, Cuenca AG, Ungaro R, Baslanti TO, McKinley BA, Bihorac A, Cuschieri J, Maier RV, Moore FA, Leeuwenburgh C, Baker HV, Moldawer LL, Efron PA (2014). A better understanding of why murine models of trauma do not recapitulate the human syndrome. Crit Care Med.

