# Supplementary figures and images for: Why do they die? Comparison of selected aspects of organ injury and dysfunction in mice surviving and dying in acute abdominal sepsis
Source: Intensive Care Med Exp. 2015 Apr 7;3:12. doi: 10.1186/s40635-015-0048-z (PMC4513036; doi:10.1186/s40635-015-0048-z)

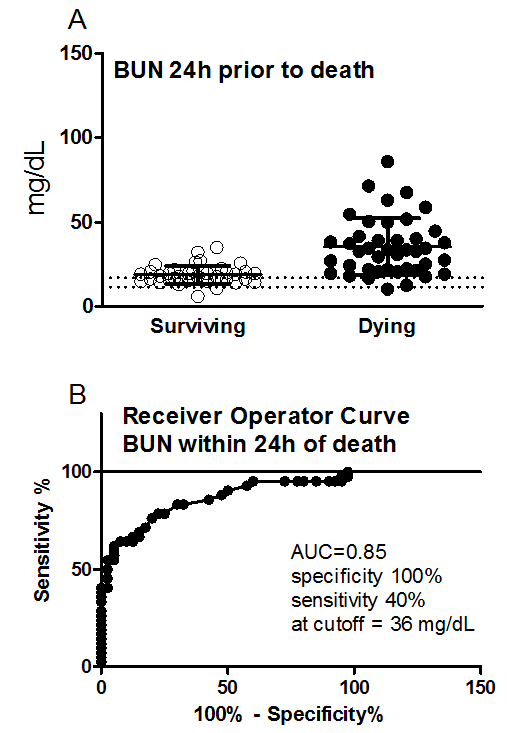

Supplement: Additional file 1: Figure S1. — Comparison of BUN elevation between dying and surviving CLP mice. (A) Plotted dots represent blood urea nitrogen (BUN) values taken from mice within 24 h of death (occurring anytime between days 1 and 4 post-CLP; right scatter) and from mice that lived until day 28 post-CLP (left scatter). Surviving n = 40; dying n = 42. (B) Predictive capacity of BUN for outcome by ROC curve assessed by measurements taken within 24 h of death in the acute phase of CLP sepsis (days 1 to 4 post-CLP). Each dot represents a single mouse. Surviving n = 40; dying n = 42. Solid horizontal lines represent mean ± SD. The two dotted horizontal lines represent 95% CI. *p < 0.05. [file 40635_2015_48_MOESM1_ESM.tiff]

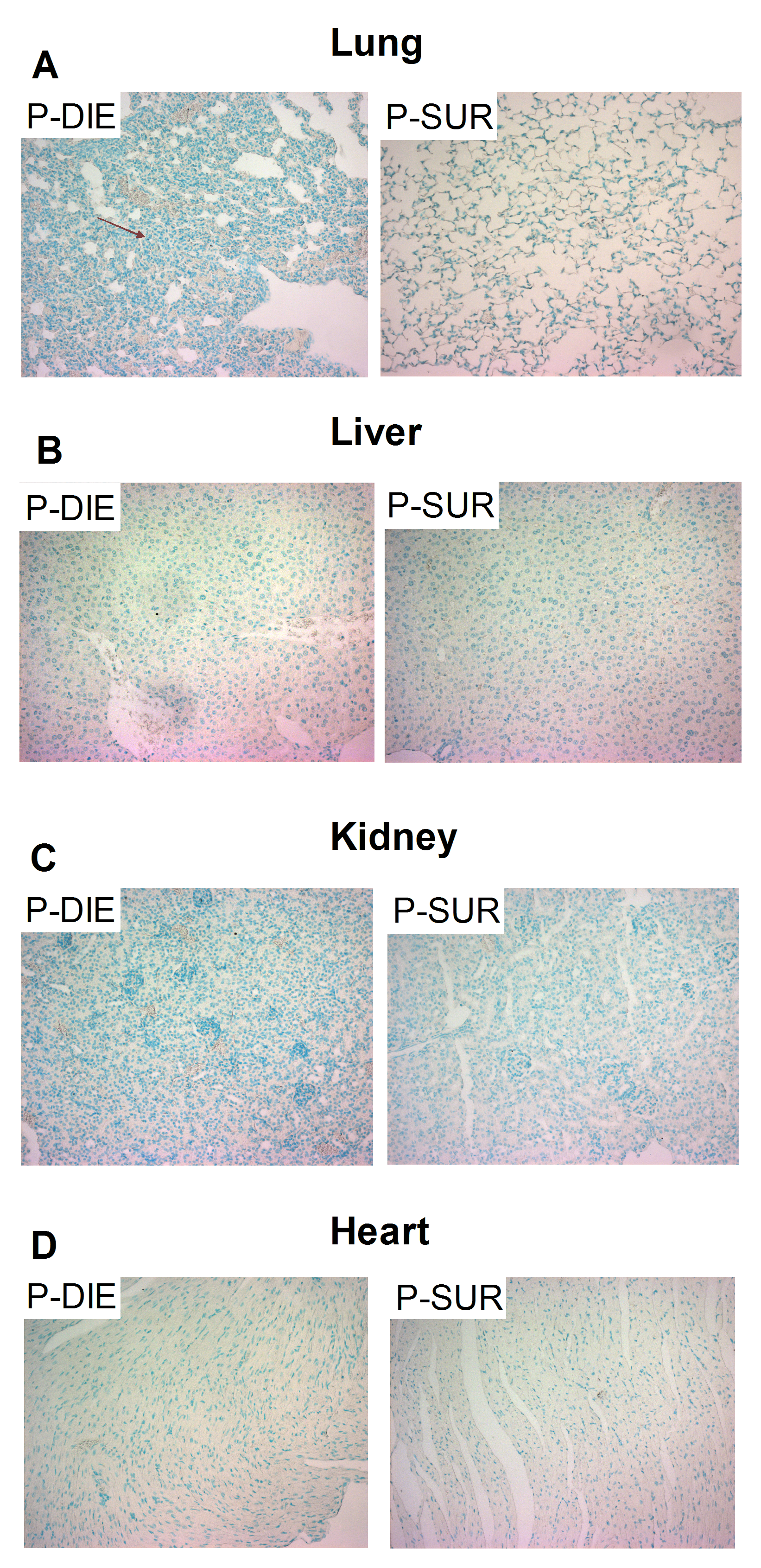

Supplement: Additional file 2: Figure S2. — Comparison of apoptosis in different organs between dying and surviving septic mice using prospective stratification of outcome. Mice were subjected to CLP, monitored for BT and stratified into either predicted to die (P-DIE) or predicted to survive (P-SUR). Upon identification, P-DIE and P-SUR mice were always sacrificed in pairs (days 1 to 3 post-CLP) and additionally compared to the healthy animals (CTRL). TUNEL-staining of (A) lung, (B) liver, (C) kidney and (D) heart was performed. P-DIE and P-SUR n = 11 and CTRL n = 7. Representative photographs are shown (original magnification ×10). Arrow in (A) indicates an infrequent apoptotic cell. [file 40635_2015_48_MOESM2_ESM.tiff]
